# Impaired STING Activation Due to a Variant in the E3 Ubiquitin Ligase AMFR in a Patient with Severe VZV Infection and Hemophagocytic Lymphohistiocytosis

**DOI:** 10.1007/s10875-024-01653-5

**Published:** 2024-01-26

**Authors:** Michelle Mølgaard Thomsen, Morten Kelder Skouboe, Michelle Møhlenberg, Jian Zhao, Kerstin de Keukeleere, Johanna Laura Heinz, Marvin Werner, Anne Kruse Hollensen, Jonas Lønskov, Ian Nielsen, Madalina Elena Carter-Timofte, Baocun Zhang, Jacob Giehm Mikkelsen, Niels Fisker, Søren R. Paludan, Kristian Assing, Trine H. Mogensen

**Affiliations:** 1 Department of Infectious Diseases, Aarhus University Hospital, Palle Juul-Jensens, Boulevard 99, 8200 Aarhus, Denmark; 2 Department of Biomedicine, Aarhus University, Aarhus, Denmark; 3 Department of Pediatrics, Odense University Hospital, Odense, Denmark; 4 Department of Clinical Immunology, Odense University Hospital, Odense, Denmark

**Keywords:** VZV, Interferon, AMFR, Ubiquitin ligase, STING, ISG, HLH

## Abstract

Varicella zoster virus (VZV) is a neurotropic *alphaherpesvirus* exclusively infecting humans, causing two distinct pathologies: varicella (chickenpox) upon primary infection and herpes zoster (shingles) following reactivation. In susceptible individuals, VZV can give rise to more severe clinical manifestations, including disseminated infection, pneumonitis, encephalitis, and vasculopathy with stroke. Here, we describe a 3-year-old boy in whom varicella followed a complicated course with thrombocytopenia, hemorrhagic and necrotic lesions, pneumonitis, and intermittent encephalopathy. Hemophagocytic lymphohistiocytosis (HLH) was strongly suspected and as the condition deteriorated, HLH therapy was initiated. Although the clinical condition improved, longstanding hemophagocytosis followed despite therapy. We found that the patient carries a rare monoallelic variant in *autocrine motility factor receptor* (*AMFR*), encoding a ubiquitin ligase involved in innate cytosolic DNA sensing and interferon (IFN) production through the cyclic GMP-AMP synthase–stimulator of IFN genes (cGAS-STING) pathway. Peripheral blood mononuclear cells (PBMCs) from the patient exhibited impaired signaling downstream of STING in response dsDNA and 2'3'-cGAMP, agonists of cGAS and STING, respectively, and fibroblasts from the patient showed impaired type I IFN responses and significantly increased VZV replication. Overexpression of the variant AMFR R594C resulted in decreased K27-linked STING ubiquitination compared to WT AMFR. Moreover, ImageStream technology revealed reduced STING trafficking from ER to Golgi in cells expressing the patient *AMFR R594C* variant. This was supported by a dose-dependent dominant negative effect of expression of the patient AMFR variant as measured by IFN-β reporter gene assay. Finally, lentiviral transduction with WT *AMFR* partially reconstituted 2'3'-cGAMP-induced STING-mediated signaling and ISG expression in patient PBMCs. This work links defective AMFR-STING signaling to severe VZV disease and hyperinflammation and suggests a direct role for cGAS-STING in the control of viral infections in humans. In conclusion, we describe a novel genetic etiology of severe VZV disease in childhood, also representing the first inborn error of immunity related to a defect in the cGAS-STING pathway.

**Supplementary Information:**

The online version contains supplementary material available at 10.1007/s10875-024-01653-5.

## Introduction

Varicella zoster virus (VZV) is a neurotropic *alphaherpesvirus* exclusively infecting humans, in whom it causes two distinct pathologies: varicella (chickenpox) upon primary infection and herpes zoster (shingles) following reactivation [1]. VZV spreads via inhalation of infectious droplets and infects mononuclear cells in the tonsils, leading to viremia through infected T cells and dissemination to the skin to replicate and cause the characteristic vesicular eruptions known as varicella [1]. Importantly, VZV establishes latency in sensory neuronal ganglia, from which it can reactivate and spread to the skin as zoster or to the central nervous system (CNS), manifesting as meningitis, encephalitis, cerebellitis, or vasculopathy with stroke [2, 3]. About 20% of patients hospitalized with chickenpox experience neurological complications [4], suggesting a correlation between impaired systemic control of the infection and development of severe varicella with dissemination to the CNS. Knowledge on the pathogenesis of VZV infection and immunity remains incomplete, and although implementation of a VZV vaccine with the Oka strain in certain countries has resulted in reduced frequency of VZV cases, this infectious disease remains a worldwide health problem [5].

VZV is an enveloped DNA virus, which can infect epithelial cells, peripheral blood mononuclear cells (PBMCs), and neurons [1, 6]. The genome of VZV comprises 71 known *open reading frames* (*ORF*)*s*, of which the *VZV latency-associated transcript* (*VLT*) is the only viral gene known to be transcribed during latency, together with a *VLT-ORF63* fusion transcript [7, 8]. Unrelated clinical VZV isolates exhibit only little variability in virulence [9], suggesting that defective host immunity is the major determinant of disease severity rather than differences between virus strains. The precise determinants of protective immunity toward VZV are incompletely understood, in part because VZV is a strictly human pathogen, and limited data are available from humanized mice studies [10]. Studies regarding inborn errors of immunity (IEI) that cause increased susceptibility to VZV are therefore of particular importance. From these studies, it is clear that cellular immunity mediated by natural killer (NK) cells and T cells plays a particularly important role during VZV infection, as individuals with IEI affecting NK and T cell function may present with severe and disseminated VZV infections [11–13]. Moreover, genetic defects in the interferon-γ-receptor 1 (IFNGR1) and tyrosine kinase (TYK) 2 interfere with macrophage function and were found to predispose to disseminated infection with mycobacteria and VZV [14, 15]. Finally, genetic defects in the innate cytosolic DNA sensor RNA polymerase III (POL III) were identified and demonstrated to cause selectively increased susceptibility to VZV CNS infection and pneumonitis in otherwise healthy children and adults [16–18].

The innate immune system utilizes pattern recognition receptors (PRRs) to detect pathogen-associated molecular patterns (PAMPs) in order to mount protective immune responses. This includes the production of cytokines and interferons (IFN)s, the latter exhibiting antiviral activity through their ability to induce IFN-stimulated genes (ISGs) [19]. Different classes of PRRs are involved in the recognition of virus infections, including membrane-associated Toll-like receptors (TLR)s, cytosolic RNA-sensing retinoic acid-inducible gene 1 (RIG-I)-like receptors, and finally cytosolic DNA sensors [19]. Within the group of DNA sensors, TLR9 detects unmethylated DNA, RNA polymerase III (POL III) recognizes AT-rich DNA, while absent in melanoma (AIM)2, gamma-interferon-inducible protein 16 (IFI16) and cyclic GMP-AMP synthase (cGAS) senses double-stranded (ds) DNA in a sequence-independent manner [20, 21]. Among these, cGAS has emerged as the main cytosolic DNA sensor, signaling through the adaptor molecule Stimulator of Interferon Genes (STING) to induce mainly type 1 IFN responses, but also to contribute to nuclear factor kappa B (NF-κB) activation [22].

Recent reports have described the occurrence of hemophagocytic lymphohistiocytosis (HLH) during VZV infection in the setting of IEI [23]. HLH is a rare but life-threatening clinical syndrome characterized by uncontrolled activation of immune cells and severe systemic inflammation [24]. Familial (or primary) HLH (fHLH) is a genetic disorder most often caused by autosomal recessive loss of function mutations in genes that disrupt the cytotoxic activity of NK and CD8 + T cells, including *PRF1*, *UNC13D*, *STX11*, *STXBP2*, *RAB27A*, *LYST*, *AP3B1*, *SH2D1A*, and *BIRC4* [25]. While autosomal recessive primary HLH presents early in childhood, hypomorphic mutations in HLH genes are generally associated with fHLH onset in adulthood [26]. Sporadic (or secondary) HLH develops without a known underlying genetic cause but rather presents in association with malignancies, autoimmune or autoinflammatory diseases, or is triggered by infections [27, 28]. Interestingly, both familial and sporadic HLH might present with an infectious trigger, and herpesviruses are the most common causes of virus-induced HLH, with Epstein-Barr-virus (EBV) and cytomegalovirus (CMV) accounting for more than 50% of virus-associated HLH [27]. Similarly, VZV can trigger HLH upon primary infection or reactivation, although this is mostly reported in patients with underlying immunosuppression or fHLH [23, 29–31].

Here, we describe a patient with severe clinical manifestations of VZV infection and HLH, whom we studied in great detail in order to understand the underlying genetic and immunological pathogenesis. We hypothesize that severe VZV disease in some otherwise healthy individuals may be caused by monogenic IEI, not necessarily displaying complete clinical penetrance. Based on the current knowledge on VZV infection pathogenesis, innate and adaptive immune circuits, and HLH-associated molecules, we searched whole exome sequencing (WES) data for rare gene variants that may underlie susceptibility to severe VZV infection and/or HLH. In this patient, we identified a monoallelic variant in the gene encoding the ubiquitin ligase autocrine motility factor receptor (AMFR) which regulates cGAS-STING signaling through STING poly-ubiquitination. This finding was functionally validated in the by showing reduced STING activation and IFN responses as well as increased VZV replication in patient PBMCs and fibroblasts from the patient compared to controls. We propose that defects in AMFR, which impair cGAS-STING-mediated sensing of foreign DNA, confer enhanced susceptibility to severe disseminated VZV infection and risk of secondary hyperinflammation.

## Methods

### Patient Material

Whole blood was collected at hospitalization in EDTA-stabilized tubes for DNA purification and after recovery in lithium heparin tubes for peripheral blood mononuclear cell (PBMC) isolation. PBMCs were isolated by Ficoll density gradient centrifugation using SepMate PBMC isolation tubes (STEMCELL Technologies, no. 85460) and frozen down in liquid nitrogen. Control PBMCs were purified from healthy donors after obtaining written consent. Genomic DNA was purified using QIAamp DNA Blood Mini Kit (Qiagen, no. 51104) according to the manufacturer’s instructions.

### WES and Bioinformatics

WES was conducted on genomic DNA from the patient using KAPA HTP library preparation and Nimblegen SeqCap EZ MedExome Plus kits and analyzed with Nextseq version2 chemistry (2 × 150 basepairs) (Ilumina). Single nucleotide polymorphisms were called relative to hg19. Variant call files were uploaded to Ingenuity Variant Analysis (IVA) software (Qiagen) and filtered according to rarity (gnomAD frequency < 0.1%) and predicted deleteriousness (Combined Annotation Depletion Dependent (CADD) score > 15 and CADD score > mutation significance cutoff (MSC) score). De novo variants were also included. In addition, variants were filtered based on their biological relevance using gene lists, including all genes related to known IEI according to the IUIS Guidelines [32] and broad biological filters in IVA related to VZV, HLH, PID, and immune response.

### Sanger Sequencing of AMFR

Genomic AMFR DNA from the patient and the patient’s mother, father, younger sister, and younger brother were amplified by PCR using Phusion Hot Start II DNA polymerase (Thermo Fischer Scientific, no. F-594S) and the following primers: AMFR forward: 5′AAGCTGCTGCTCCATTATCCG-3′ and AMFR reverse 5′-TACCAGCATCAGAGGTAGACCA-3′. The genotype of amplified AMFR was confirmed using Sanger sequencing with AMFR forward primer.

### In Vitro Stimulations of PBMCs

Patient and control PBMCs were thawed and seeded in RPMI supplemented with 10% heat-inactivated fetal bovine serum and 1% penicillin/streptomycin (cRPMI) at a density of 5 × 10^5^ cells/well for infection experiments and 1 × 10^6^ cells/well for stimulation with 2′3′-cGAMP and dsDNA and incubated overnight at 37 °C and 5% CO_2_. Cells were infected with 50 HAU of Sendai virus (SeV) (Cantell strain, Charles River), 3 MOI of HSV1 (KOS Strain), or 0.2 MOI of cell-free (CF) VZV debris (rOKa strain) or CF debris mock). Following 24 h of infection, supernatants were harvested for mesoscale, and cells were lysed for RNA isolation. In addition, PBMCs were stimulated with 100 µg/mL of 2′3′-cGAMP (InvivoGen) or 2 µg/mL of transfected ht (herring testes) dsDNA for 3 h following cell lysis for Western blotting or stimulated with 50 µg/mL high molecular weight poly(I:C) (InvivoGen, USA) or 5 µg/mL CpG ODN2395 (InvivoGen, USA) for 6 h before being lysed for downstream RT-qPCR analysis.

### Fibroblast Experiments

A skin biopsy from P1 was taken under local anesthesia after the clinical symptoms had disappeared, with the written consent of P1 and his parents. Dermal fibroblasts were subcultured at the Department of Clinical Genetics, Aarhus University Hospital (Aarhus, Denmark). Normal human dermal fibroblasts (NHDF) were obtained from Promocell (Heidelberg, Germany). Primary fibroblasts were grown in DMEM with 10% FBS and 1% penicillin–streptomycin.

### VZV Infection

Primary fibroblasts were seeded at a density of 60,000 cells/well in 24-well plates. The following day, growth media was removed, and cell-free VZV rOka strain debris was added to the cells in 0.6 mL Hank’s Balanced Salt Solution (Gibco, Montana, USA) at MOI 1. The plate was centrifuged at 250 × g for 10 min after which the virus adsorbed for 4 h at 37 °C. The virus media was removed, and the cells were washed once in PBS before the addition of 0.5 mL 10% DMEM. At 48 hpi, the supernatant was removed, and the cells were washed once in PBS before lysing for downstream RT-qPCR measurement of viral ORF expression.

### RT-qPCR

Total RNA was purified using Nucleospin 96 RNA core kit (Macherey–Nagel, Germany). For two-step RT-qPCR, the RNA was reverse transcribed into cDNA using Iscript™ gDNA Clear cDNA Synthesis Kit (Bio-Rad, USA). qPCR was then performed with either TaqMan Fast Advanced Master Mix (Applied Biosystems, USA) or Brilliant III Ultra-Fast SYBR Green qPCR Master Mix (Agilent, USA). For one-step RT-qPCR, the TaqMan RNA to CT One Step Kit (Applied Biosystems, USA) was used. Relative mRNA was calculated using the 2^−ΔΔCq^ method.

### RT-qPCR Primers

The following TaqMan primers were used (Thermo Fisher Scientific, USA): AMFR (Hs01031688_m1), IFNB1 (Hs01077958_s1), MX1 (Hs00895608_m1), CXCL10 (Hs00171042_m1), TBP (Hs00427620_m1), GAPDH (Hs02758991_g1), and 18S (Hs03928985_g1).

The following PCR-primers were used for SYBR Green qPCR: ORF63 forward: 5′-GCGCCGGCATGATATACC-3′, ORF63 reverse: 5′-GACACGAGCCAAACCATTGTA-3′; ORF40 forward: 5′-ACTTGGTAACCGCCCTTGTG-3′; ORF40 reverse: 5′-CGGGCTACATCATCCATTCC-3′, ORF9 forward: 5′-GGGAGCAGGCGCAATTG-3′, ORF9 reverse: 5′-TTTGGTGCAGTGCTGAAGGA-3′. WPRE forward: 5′-GGCACTGACAATTCCGTGGT-3′, WPRE reverse: 5′-AGGGACGTAGCAGAAGGACG-3′, ALB forward: 5′-GCTGTCATCTCTTGTGGGCTGT-3′, and ALB reverse: 5′-ACTCATGGGAGCTGCTGGTTC-3′, PPIB forward: 5′-CAACGCAGGCAAAGACACCAAC-3′, PPIB reverse: 5′-GGTTTATCCCGGCTGTCTGTCTTG-3′.

### Western Blotting

For PMBC stimulations, cells were washed twice with PBS and lysed in RIPA buffer (Thermo Fischer Scientific, no. 89901) supplemented with Halt Protease and Phosphatase Inhibitor Cocktail (Thermo Fischer Scientific, no. 78440) and Benzonase Nuclease (Sigma-Aldrich, no. E1014-25KU). Protein concentrations were measured using Pierce BCA Protein Assay Kit (Thermo Scientific, no. 23227), and cell lysates were denatured at 95 °C for 5 min with 50 mM DTT (Sigma-Aldrich, no. 43816-10ML) and 4 × Laemmli buffer (Bio-Rad, no. 1610747). Samples were subjected to SDS gel electrophoresis and transferred to a PVDF membrane using the Transfer-Blot Turbo systems. The membrane was blocked in 5% skimmed milk in PBS-T or TBS-T for 1 h, followed by incubation overnight at 4 °C with primary antibodies against STING (cell signaling, no. 13647S, 1:1000), phospho-STING (cell signaling, no. 19781S, 1:1000), TBK1 (cell signaling, no. 3013S 1:1000), phospho-TBK1 (cell signaling, no. 5483S, 1:1000), IRF3 (cell signaling, no. 11904S, 1:1000), phospho-IRF3 (cell signaling, no. 4947S, 1:1000), ISG15 (cell signaling, no. 2758S, 1:1000), Vinculin (cell signaling, no. 13901S, 1:2000), AMFR (Proteintech, no. 16674–1-ap), GFP (Santa Cruz no. sc-8334, 1:500), FLAG-Tag (cell signaling, no. 17793S, 1:500), Myc-Tag (cell signaling, no. 2278S, 1:1000) or HA-Tag (cell signaling, no. 3724S, 1:1000). Primary antibodies were visualized using secondary horseradish-peroxidase-coupled anti-rabbit or anti-mouse antibodies (Jackson ImmunoResearch no. 715–036-150, no. 711–035-152) at 1:10.000 on ChemiDoc gel imaging system (Bio-Rad).

### Mesoscale Measurements of Cytokines and Interferons in Supernatants

Expression of IFNs (IFNα2, IFNβ, IFNλ1, and IFNγ) and proinflammatory cytokines (IL-1β and TNFα) was measured in cell culture supernatants using U-PLEX assays (Meso Scale Diagnostics, USA) according to manufacturer’s protocols on a Meso Quickplex SQ 120 instrument.

### STING Immunoprecipitation

HEK293T cells were seeded at a density of 2.5 × 106 cells in a 6-cm Petri dish and following overnight incubation, transfected with 1 µg of the following plasmids: pcDNA3/FLAG-STING, pRK5/HA-K27-ubiquitin, and pcDNA3/Myc-AMFR WT or pcDNA3/Myc-AMFR R594C. Controls transfected only with FLAG-STING, HA-K27-Ubiquitin, and pcDNA3-empty vector (No AMFR control), or HA-K27-Ubiquitin, AMFR WT, and FLAG-Empty vector (No STING control) were included. Cells were lysed 24 h post transfection in Pierce IP lysis buffer (Thermo Fischer Scientific, no. 8788) supplemented with 1 × protease inhibitor cocktail (Roche, no. 05.892.970.001) and 1 × PhosSTOP cocktail (Roche, no. 04.906.837.001) for 1 h at 4 °C with rotation and centrifuged for 10 min at 1400 × g at 4 °C. Lysates were then incubated with FLAG M2 Dynabeads (Sigma, no. M8823) for 1.5 h at room temperature, washed 3 times in TBS with 0.05% Tween20 and phosphatase inhibitors (TBS-T +) and incubated with 3XFLAG peptide (Sigma-Aldrich) for 15 min at room temperature to elute STING. Then, eluents were denatured by boiling for 5 min at 95 °C in the presence of 1% sodium dodecyl sulfate (SDS) after which samples were diluted in lysis buffer with protease-, phosphatase-, and deubiquitylating enzyme inhibitors (Merck, no. 662141) and incubated overnight at 4 °C with FLAG M2 Dynabeads. Finally, samples were washed three times in TBS-T and FLAG-STING eluted by boiling the samples in Laemmli concentrate sample buffer (Sigma, no. S3401-10VL) at 95 °C for 10 min. Lastly, samples were immunoblotted for expression of FLAG-STING, Myc-AMFR, and HA-Ubiquitin.

### IFNB Luciferase Reporter Gene Assay

HEK293T cells with stable STING expression were plated on 96-well plates (20–30.000 cells per well) and transfected with 30 ng constructs harboring *IFNB*-promoter firefly luciferase reporter genes, and 15 ng β-actin-promoter-driven Renilla luciferase together with the indicated amounts of the *AMFR WT* and *AMFR R594C*. At 18 h post-transfection, the cells were stimulated with 2′3′-cGAMP or left untreated. After 8 h of stimulation, the cells were lysed, and the Firefly and Renilla luciferase signals were developed with a Dual-Glo luciferase assay (Promega) and read on a Luminoskan Ascent (Labsystems) according to the manufacturer’s instructions.

### ImageStream

The co-localization of STING–ER and STING–Golgi, was determined by ImageStream using the ImageStream MK II Imaging Flow Cytometer (Amnis). The cells transfected with the indicated plasmids for 24 h were fixed using 4% formalin for 20 min at room temperature and then pre-permeabilized with 0.2% Triton X-100 for 6 min. The cells were incubated with primary antibodies for 1 h on ice and then incubated with the Alexa-Fluor-labeled secondary antibodies for 1 h. After every step, the cells were washed with 1 × PBS three times. Finally, the cells were resuspended in 1 × PBS with 2 mM EDTA and 3% BSA. An original magnification of × 60 was used for all samples. The images of 7000–10,000 single cells with different channels were acquired in the ImageStream, and the data were analyzed through IDEAS software v6.2 (Amnis Corporation). The antibodies used were mouse anti‐STING (1:300), rabbit anti-PDI (1:50), and rabbit anti-GM130 (1:3000). After gating of focused, single and positive cells with the defined fluorescent markers, the colocalization of STING–PDI (ER marker) and STING–GM130 (Golgi marker) was analyzed through the Bright Detail Similarity feature for the corresponding channels. The accuracy of the cell populations gated as representing colocalization was controlled by visual inspection of individual pictures in the gated cell populations.

### Lentiviral Reconstitution of Patient PBMCs

Third-generation lentiviral vectors were produced as previously described [33]. Briefly, HEK293T cells were transfected with 11.25 µg pMD.2G, 9 µg pRSV-Rev, 39 µg pMDLg/pRRE, and 39 µg lentiviral transfer vector for T175 bottles using a standard polyethylenimine transfection protocol. Five hours after transfection, the medium was changed. Forty-eight hours after transfection, viral supernatants were harvested, filtered, and ultracentrifuged at 25,000 rcf for 2 h using a sucrose gradient (sucrose, 20% w/v) before titration by quantification of the number of lentiviral integrations in HEK293T as previously described [33]. For the reconstitution, patient and control PBMCs were thawed and seeded as described above. At the time of seeding, the cells were stimulated with 150 1.5 µg/ml PHA (Fisher Scientific, USA) for 72 h before transduction with MOI 10 of lentiviral particles. Seventy-two hours after transduction cells were stimulated with 2′3′-cGAMP as previously described, followed by cell lysis for Western blotting.

### Statistics

For statistical testing, Prism 9.2 (GraphPad Software, USA) was used. All tests were two-tailed and an adjusted alpha < 0.05 was considered statistically significant. Correction for multiple comparisons was carried out as specified in the figure legends.

### Ethics

The patient, family, and healthy controls were included following oral and written consent in accordance with The Helsinki Declaration and national ethics guidelines and after approval from the Danish National Committee on Health Ethics (no. 1–10-72–275-15), the Data Protection Agency, and Institutional Review Board.

## Results

### Case Description: 3-Year-old Boy with Disseminated VZV Infection and Hemophagocytic Lymphohistiocytosis

A 3-year-old boy was admitted to hospital and diagnosed with severe and probably disseminated varicella infection with high fevers, pneumonitis, and impaired consciousness. Treatment with intravenous high-dose acyclovir and broad-spectrum antibiotics was initiated, and he was transferred to a tertiary center where extensive and progressive systemic inflammation, splenomegaly, and hemophagocytosis in the bone marrow were diagnosed. CNS infection was suspected, but a lumbar puncture performed several days into the disease course did not reveal pleocytosis or the presence of VZV in the CSF. HLH with fever, hemophagocytosis in the bone marrow, and paraclinical findings fulfilling the HLH2004 criteria were diagnosed and treated according to protocol. WES was performed and initially analyzed for presence of variants in a gene panel consisting of 291 PID and HLH-related genes, but no known disease-causing mutations were identified, and immunological analysis also did not demonstrate any significant abnormalities in cytotoxic NK cell or T cell function. Despite reasonable response to treatment, the boy experienced long-standing inflammation with elevated inflammatory markers and persistent finding of activated macrophages with hemophagocytosis in the bone marrow, until after 40 weeks at which point therapy could finally be discontinued; when reexamined 4 months later, hemophagocytosis had disappeared. Aciclovir prophylaxis was discontinued 16 months after the initial infection, and the boy finally recovered and is presently without any infections or inflammation at the age of 8 years. However, based on the severe and prolonged infectious and inflammatory disease course, we hypothesize that the patient may have an unrecognized IEI predisposing to severe VZV infection, which in turn, led to the development of hyperinflammation and secondary HLH. Serological analyses showed that the patient was anti-VZV and anti-HSV 1/2 negative as of March 2017, whereas the mother was anti-VZV positive in 2017. Two younger siblings were both VZV vaccinated with an initial dose at age 9 months; HSV and VZV serology do not exist for these at the time of writing.

### Identification of a Variant in the E3 Ubiquitin Ligase AMFR Predicted to be Deleterious

**Fig. 1:**
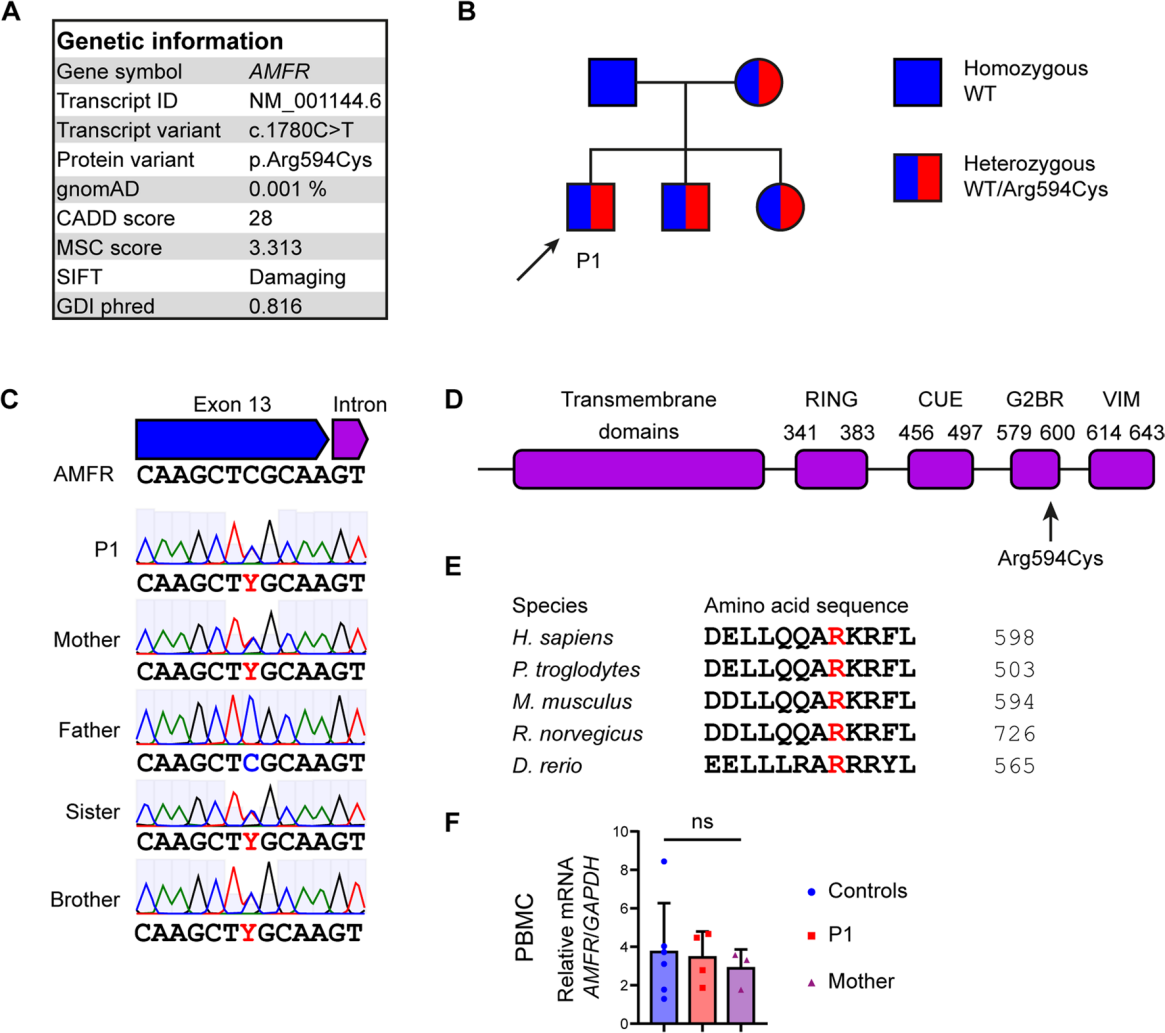
Identification of a rare genetic variant in *AMFR* in a patient with severe VZV infection and HLH. **A** Characteristics of the monoallelic *AMFR* variant. **B** Pedigree showing heterozygous inheritance of the *AMFR R594C* variant inherited from the mother. **C** Sanger sequencing confirming the presence of the *AMFR R594C* variant in the patient (P1), the patient’s mother, and siblings. **D** Protein structure of AMFR with localization of the R594C variant in the G2BR domain. **E** Protein alignment showing conservation of Arginine at position 594 in AMFR across different species. **F** AMFR mRNA levels were measured using RT-qPCR of whole-cell lysates of PBMCs from healthy controls, P1, and his mother. Statistics was done by ordinary 1-way ANOVA. not significant (ns). CADD, combined annotation dependent depletion; MSC, mutation significance cutoff; SIFT, sorting intolerant from tolerant; gnomAD, the genome aggregation database; GDI, Gene Damage Index; RING, really interesting new gene; CUE, coupling of ubiquitin conjugation to ER degradation; G2BR, Ube2g2-binding region; VIM, p97/VCP-interacting motif

As the initial diagnostic sequencing analysis did not identify any mutations within a panel of known PID and HLH-related genes, we went on and performed a full and unbiased WES to look for the presence of a rare genetic variant that might have contributed to disease in the patient. We based the analysis on a hypothesis of autosomal dominant (AD) inheritance with possible incomplete penetrance or de novo mutations but also examined rare bi-allelic variants. During the analysis, variants were kept if they were rare (frequency < 0.1%), predicted to be pathogenic as defined by a CADD score > 15 and above the mutation significance cutoff (MSC) score, and had relevance to antiviral immunity and/or inflammation. Remaining variants (Supplementary Table 1) were manually inspected to establish the biological function of each variant-encoding gene. In this process, the exome was also examined for all known genes related to IEI (according to IUIS guidelines 2023 [32]), of which none was identified. This approach led to the identification of a rare, monoallelic missense variant in the *AMFR* gene, predicted to be deleterious by various bioinformatic prediction tools, including polymorphism phenotyping v2 (PolyPhen-2), sorting intolerant from tolerant (SIFT), and CADD. In addition, the gene damage index (GDI) Phred score indicated low tolerance for damaging loss of functional variants within the *AMFR* gene (Fig. 1A). The variant was confirmed by Sanger sequencing of patient genomic DNA (Fig. 1B), and relatives were also Sanger sequenced revealing the presence of the variant in the infant sister and in the brother and mother, suggesting AD inheritance with incomplete penetrance (Fig. 1B–C). The variant, *AMFR* c.1780C > T, results in an amino acid substitution of the highly conserved arginine to cysteine residue at position 594 within the G2BR domain of the molecule (Fig. 1A and D–E). AMFR/gp78 is an E3 protein ubiquitin ligase embedded in the endoplasmic reticulum (ER) membrane, important in the degradation of misfolded proteins in the ER-associated degradation (ERAD) process [34].

The protein consists of five N-terminal transmembrane domains, a RING domain, a Cue domain and C terminal G2BR, and VIM domains (Fig. 1D). Upon microbial DNA challenge, AMFR catalyzes K27-linked polyubiquitination of STING, and this ubiquitin chain creates an anchoring platform for recruiting and activating serine/threonine-protein kinase (TBK)1, resulting in IFN regulatory factor (IRF)3 phosphorylation and IFN-β induction [35]. These data together suggested a potentially disease-causing role of the identified AMFR variant and prompted further functional analyses for functional validation and establishment of the molecular mechanism involved in increased susceptibility to VZV infection and development of HLH in the patient.

### Patient Cells Exhibit Reduced Signaling Downstream of STING

**Fig. 2:**
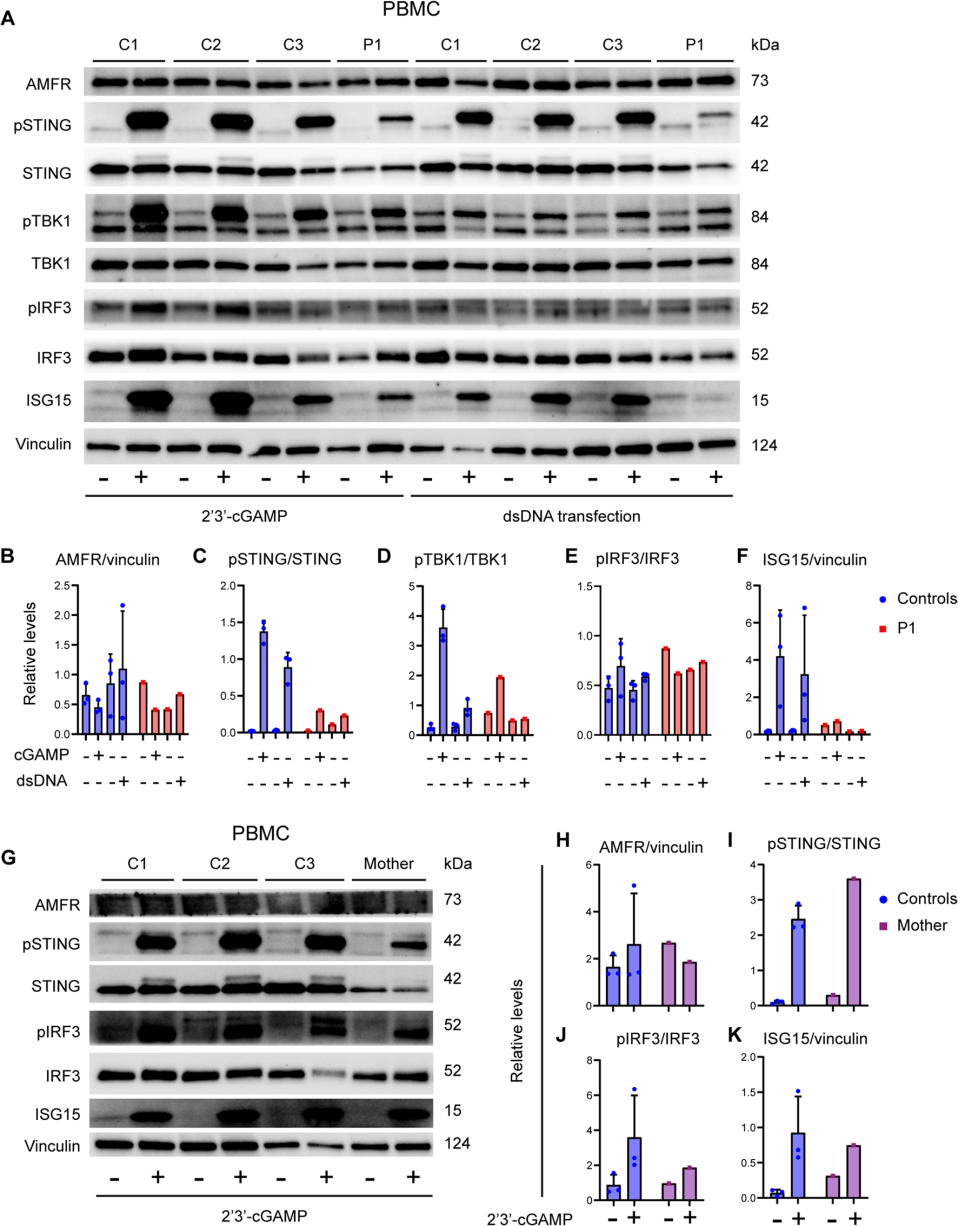
Reduced STING signaling and ISG responses in patient cells. **A** PBMCs from the patient and three healthy controls (C1–C3) were stimulated with 100 µg/mL of 2′3′-cGAMP (**A**) or 2 µg/mL transfected dsDNA for 3 h and lysates were subjected to western blotting for the expression levels of pSTING, STING, pTBK1, TBK1, pIRF3, IRF3, ISG15, and vinculin (loading control) **B–F** Quantification of the intensity of the western blot bands in (**B**). **G** PBMCs from the mother and three healthy controls were stimulated with 100 µg/mL of 2′3′-cGAMP for 3 h. Lysates were subjected to western blotting for the protein expression of pSTING, STING, pIRF3, IRF3, ISG15, AMFR, and vinculin (loading control). **H–K** Quantification of intensity of the western blot bands in (**G**)

We first investigated STING-related signaling pathways in patient cells. First, we observed comparable expression of AMFR mRNA (Fig. 1F) and protein in patient PBMCs and controls (Fig. 2A, B), suggesting that the functional defect observed in patient PBMCs might be due to altered functionality rather than decreased expression of AMFR. We stimulated patient PBMCs with 2′3′-cGAMP or transfected dsDNA for 3 h and performed immunoblotting on whole cell lysates. This revealed markedly reduced phosphorylation of STING and the downstream kinase TBK1 in patient PBMCs compared to controls (Fig. 2A–F and Supplementary Figure S1). Importantly, induction of ISG15 protein was clearly reduced in patient PBMCs (Fig. 2A, F). Similar experiments immunoblotting 2′3′-cGAMP-stimulated cell lysates from the mother demonstrated reduced phosphorylation of IRF3 but with STING phosphorylation and ISG15 expression comparable to controls (Fig. 2G–K).

**Fig. 3:**
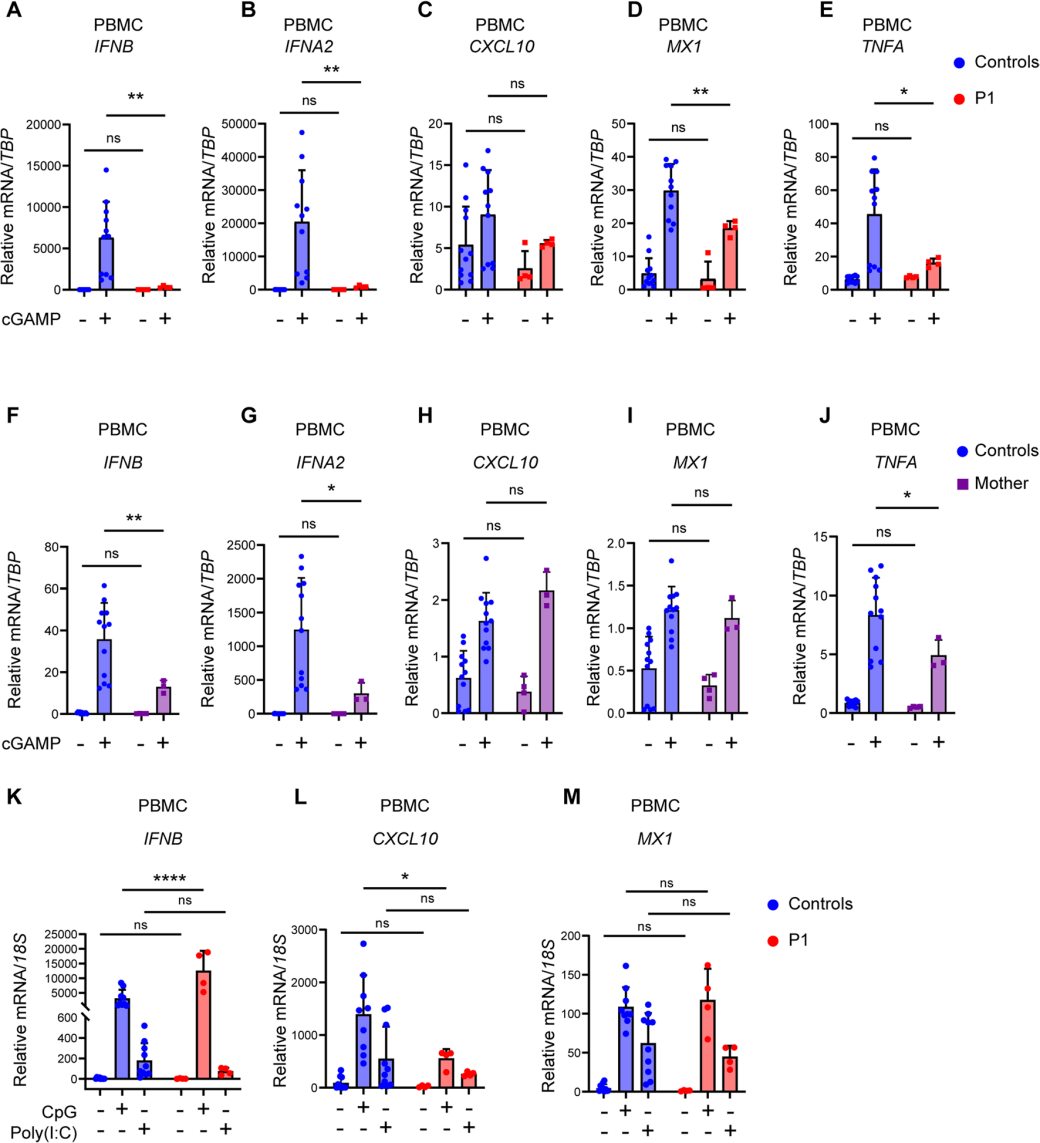
Impaired cGAMP-induced responses and preserved TLR3 and TLR9 signaling in patient cells. PBMC from healthy controls and P1 (**A–E**), or his mother (**F–J**), were stimulated with 100 µg/mL 2′3′-cGAMP for 6 h. Total RNA was isolated and mRNA levels of IFNB, IFNA2, CXCL10, MX1, and TNFA were measured relative to TBP. **K–M** PBMCs from P1 and three healthy controls were stimulated with poly(I:C) (50 µg/mL) or CpG ODN2395 (5 µg/mL) for 6h, after which total RNA was isolated and levels of *IFNB1*, *CXCL10*, and *MX1* were measured relative to *18S* mRNA. Statistics: 2-way ANOVA with Sidak’s multiple comparisons test. Ns, non-significant; *p ≤ 0.05, **p < 0.01, ****p ≤ 0.0001

In accordance with the above results suggesting disturbed STING signaling in patient cells, stimulation of PBMCs with 2'3'-cGAMP revealed significantly reduced expression of IFNa, IFNβ, MX1, and TNFα in patient PBMCs compared to controls (Fig. 3Α–Ε). Similar to the patient, the mother’s PBMCs also showed decreased production of IFNα, IFNβ, and TNFα in response to 2′3′-cGAMP, although expression of ISGs was not decreased (Fig. 3F–J). As a control, stimulation of patient PBMCs with poly(I:C) or CpG showed no defect in IFNα, CXCL10, and MX1 mRNA induction in P1, demonstrating the integrity of the TLR3 and TLR9 pathways, respectively (Fig. 3K–M). Since no increased ISG signature was observed neither in unstimulated nor in stimulated patient cells, these data do not suggest the presence of an interferonopathy.

### Increased VZV Replication in Patient PMBCs and Fibroblasts and Impaired Type I IFN Responses in Patient Fibroblasts

**Fig. 4:**
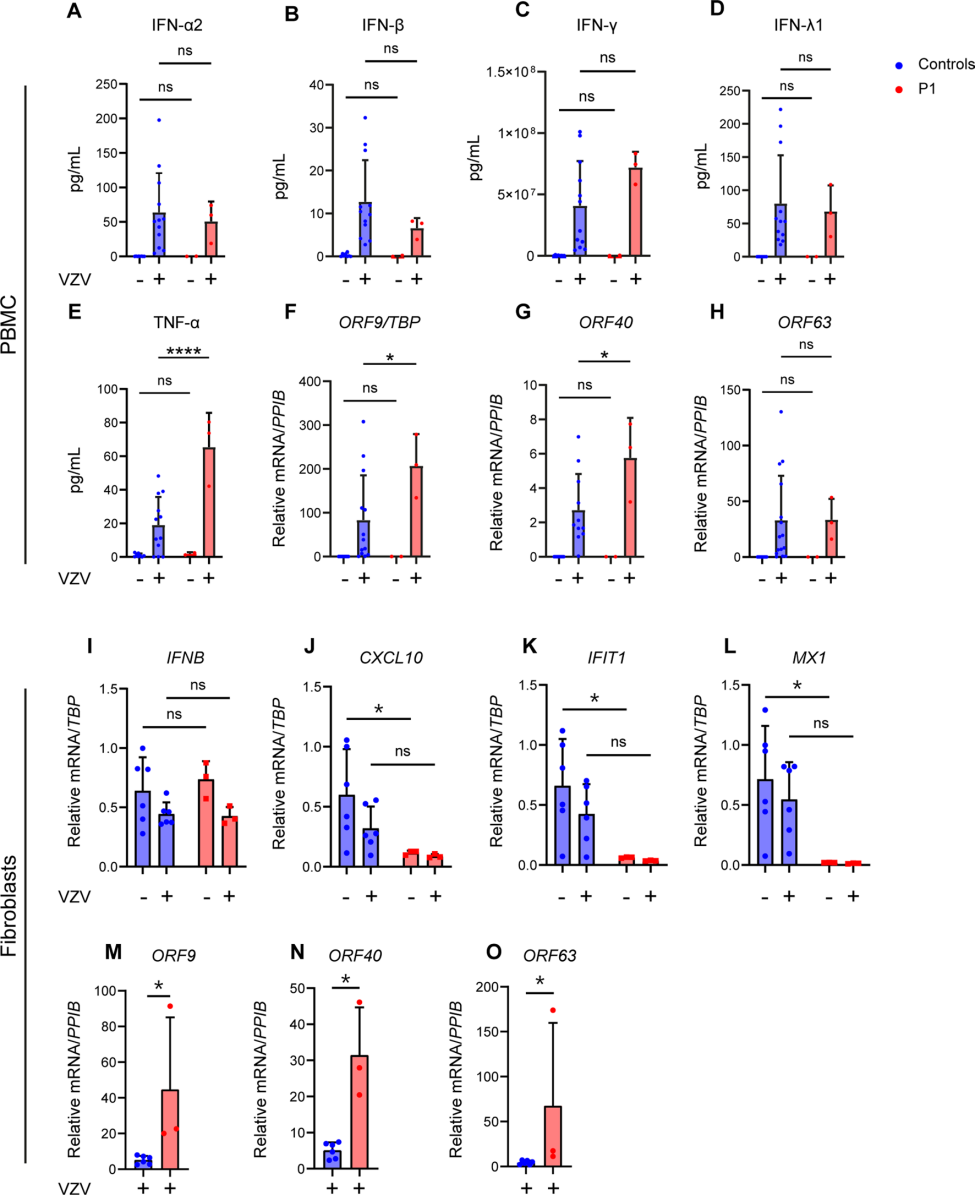
Antiviral and proinflammatory immune responses and viral replication in patient cells infected with cell-free VZV. **A–H** PBMCs from P1 and healthy controls were infected with cell-free VZV rOka vaccine-strain (VZV rOka), MOI 0.2 for 24 h. **A–F** Supernatants were examined for the level of IFN-α2, IFN-β, IFN-λ1, IFN-γ, and TNF-α using Mesoscale U-plex assays. **F–H** VZV infected PBMCs were lysed, and RNA was purified and subjected to RT-qPCR for the expression of VZV *open reading frame gene (ORF)9*, *ORF40*, and *ORF63*. *ORF* mRNA was normalized to *TBP* levels, and statistics were calculated using a 2-way ANOVA and Sidak’s multiple comparisons test. The experiment is representative of three independent experiments. **I–O** Primary fibroblasts of P1 and two healthy controls were infected with VZV rOka at MOI 1. At 48 hpi, the cells were lysed for RT-qPCR of viral *ORF* expression and ISG mRNA. **I–L** mRNA levels of *IFNB*, *CXCL10*, *IFIT1*, and *MX1* were normalized to TBP. **M–O** *ORF* mRNA levels were normalized to *PPIB*. Statistics: Mann–Whitney *U*-test. Statistics: 2-way ANOVA and Sidak’s multiple comparisons test. Abbreviations: Ns, non-significant; *p < 0.05, ****p ≤ 0.0001

To evaluate the functional impact of the AMFR variant in patient cells, we next measured antiviral and inflammatory responses in patient PBMCs (harvested when the patient was asymptomatic) infected with cell-free (CF) VZV rOka vaccine-strain in vitro. However, surprisingly we observed VZV-induced production of IFN-α2a, IFN-β, and IFN-λ1 measured by multiplex ELISA to be similar in patient and healthy controls (Fig. 4A–D), whereas TNF-α production in response to VZV infection was increased in patient PBMCs compared to healthy controls (Fig. 4E). To examine the degree of viral control in patient cells, we measured VZV open reading frame (ORF) mRNA induction in VZV-infected PBMCs using expression of these ORFs and observed significantly increased expression of the two viral genes ORF9 and ORF40 in patient cells (Fig. 4F–H). VZV stimulation of the mother’s PBMCs showed decreased IFNB mRNA expression but intact expression of IFNA2, MX1, IFIT1, and TNFA (Fig. S2A–E) together with significantly increased viral replication (Fig. S2F–H). This effect was even more striking in patient fibroblasts, in which induction of the ISGs CXCL10, IFIT1, and MX1 was significantly impaired compared to controls (Fig. 4I–L), accompanied by elevated levels of viral ORF mRNA (Fig. 4M–O).

**Fig. 5:**
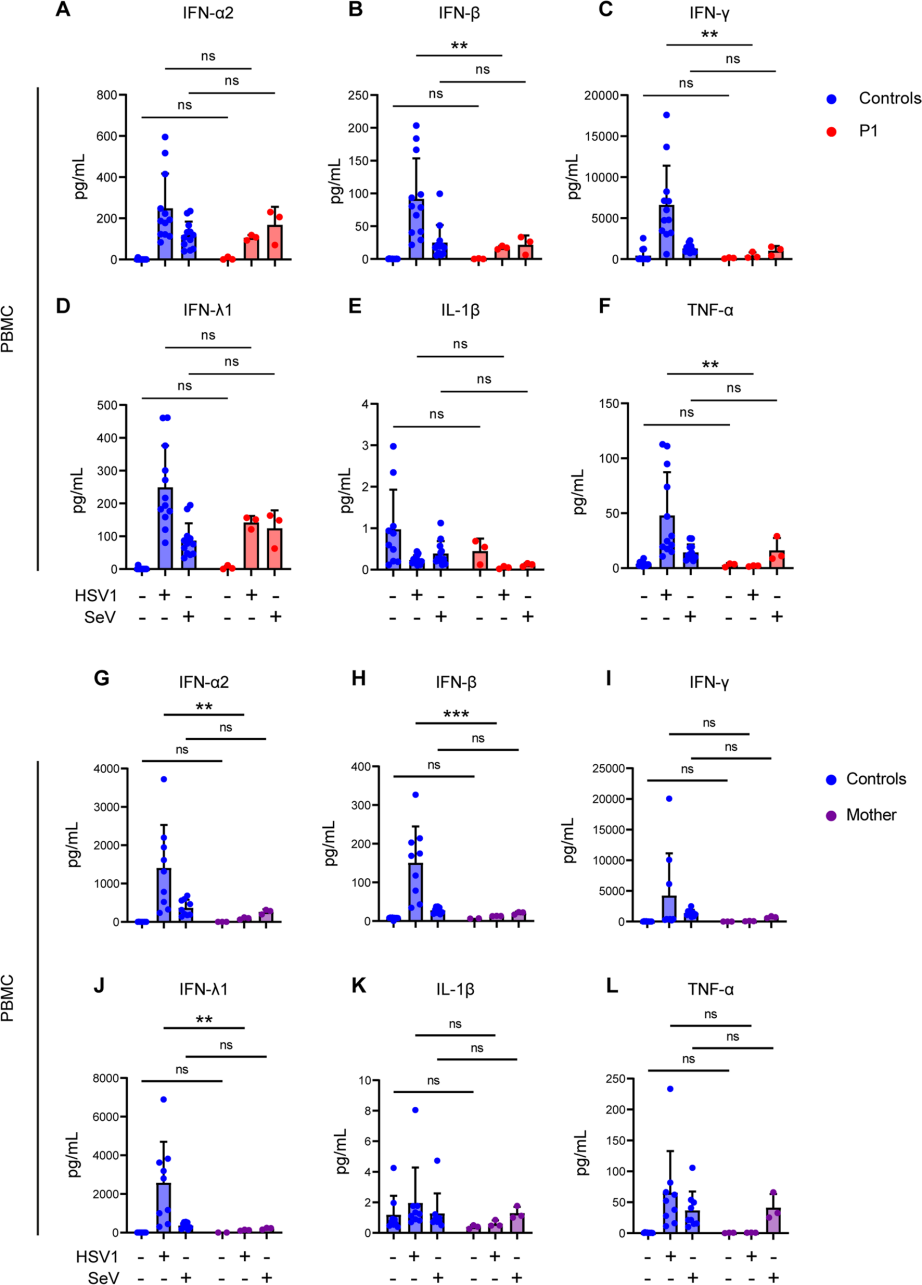
Induction of IFNs and proinflammatory cytokines in patient PBMCs in response to HSV1 and SeV infection. **A–L** PBMCs from P1 **A–F** or the mother **G–L** were infected with HSV1 (MOI 3) or SeV (50 HAU/500,000 cells), and supernatants harvested 24 h post-infection, and levels of IFN-α2, IFN-β, IFN-λ1, IFN-γ, IL-1β, and TNF-α were measured using Mesoscale U-plex assays. Statistical significance was calculated using a 2-way ANOVA and Sidak’s multiple comparisons test. Ns, non-significant; ** ≤ 0.01. The experiments were performed twice

Interestingly, when we infected patient PBMCs with the closely related *alphaherpesvirus herpes simplex virus* (HSV)-1, we found statistically significant decreased levels of IFN-α2, IFN-β, IFN-γ, and TNF-α in cell culture supernatants of patient PBMCs compared to healthy control (Fig. 5A–F). Moreover, this trend was not observed when patient PBMCs were subjected to infection with SeV, a single-stranded RNA virus, in which case patient cells responded with normal production of type I, II, and III IFNs as well as proinflammatory cytokines as compared to healthy controls (Fig. 5A–F). Similar infection experiments of PBMCs from the mother also revealed significantly decreased IFN-α2, IFN-β, and IFN-λ in response to HSV1, whereas responses to SeV were equal to controls (Fig. 5G–L). Collectively, these data indicate a specifically impaired antiviral immune response to HSV1 and possibly VZV, the latter being more difficult to ascertain due to the highly cell-associated nature of VZV. Most notably, these results demonstrate significantly increased VZV replication in patient PBMCs and fibroblasts compared to controls, thereby linking the cellular phenotype of increased virus replication and pathogen load to the clinical disease presentation consisting of severe disseminated VZV infection with possible CNS involvement, ultimately triggering HLH.

### Reduced K27-Linked STING Polyubiquitination in HEK293T Cells Expressing Mutant AMFR

**Fig. 6:**
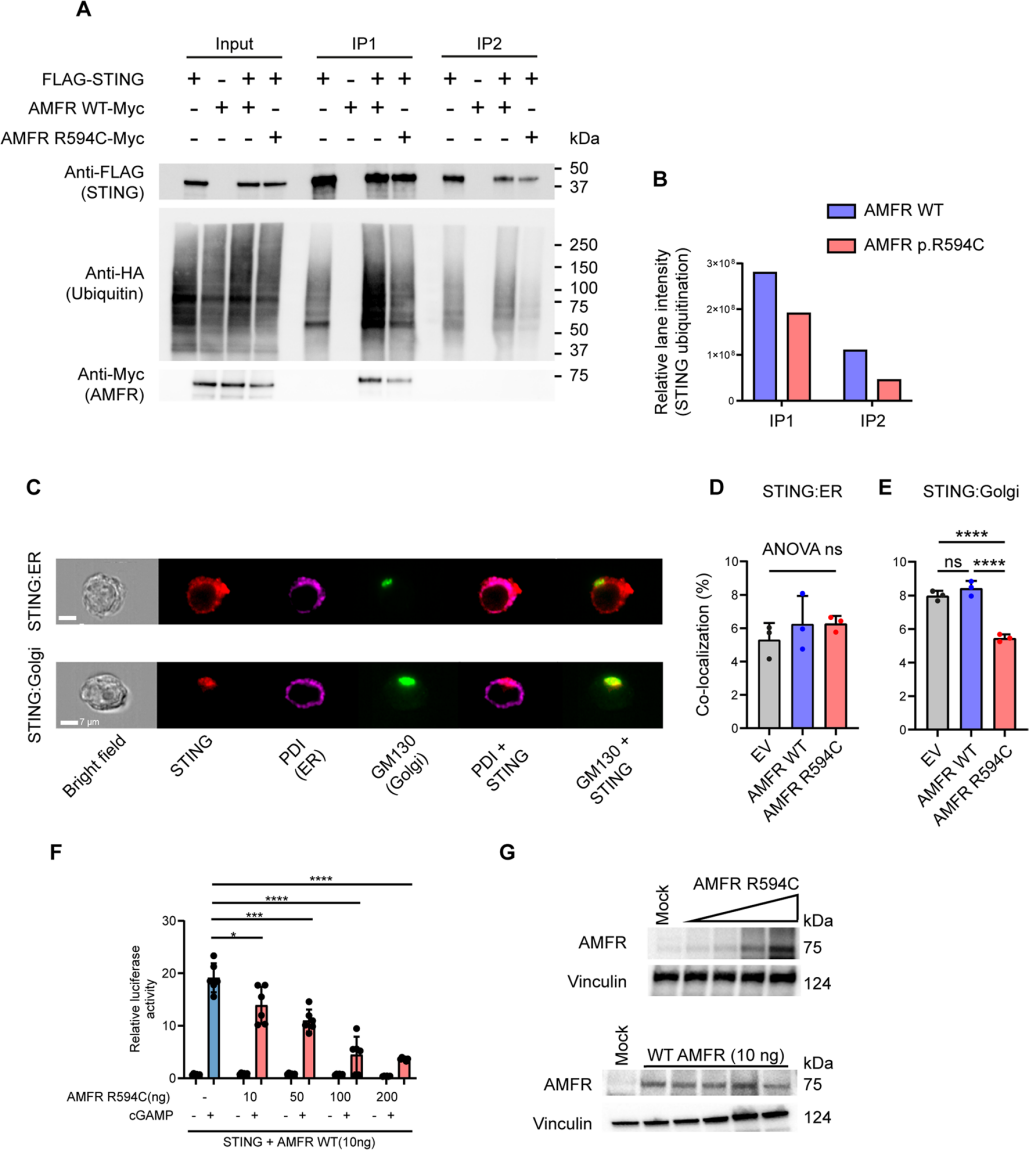
Decreased STING ubiquitination in cells expressing the patient AMFR R594C variant. **A** HEK293T cells were transfected with 1 µg of FLAG-STING, HA-K27 ubiquitin, and AMFR-Myc-WT or AMFR-Myc-R594C for 24 h, after which STING was immunoprecipitated twice with anti-FLAG-M2 beads. Lysates were immunoblotted for expression of FLAG-STING, HA-(K27) ubiquitin, and Myc-AMFR. **B** HA-ubiquitin expression was quantified in ImageLab (Bio-Rad, USA) by measuring the total lane intensity of the HA-ubiquitin smear for both AMFR-WT and AMFR-R594C mutant expressing cells in IP1 and IP2. The experiments are representative of three independent experiments. **C–D** HEK293T cells were transfected as indicated and probed with mouse anti-STING and anti-PDI (ER marker) or anti-GM130 (Golgi marker). Cells were analyzed by ImageStream. Panel **C** shows representative images, and panels **D **and** E** show quantification of STING-ER colocalization and STING-Golgi colocalization from three independent experiments of STING-ER co-localization **F**, **G** HEK 293T cells with stable STING expression were transfected with 10, 50, 100, 200 ng AMFR R594C or empty vector, IFNB1 promoter luciferase reporter, and β-actin Renilla *reporter.* **F** Eighteen hours after transfection, the cells were stimulated with 2′3′-cGAMP (50 mg/mL) for 8 h. **G** Cells were lysed for western blotting for AMFR and vinculin (loading control). Statistics were calculated with a 2-way ANOVA and Dunnett’s multiple comparisons test. *p<0.05; ***p < 0.001; ****p < 0.0001. The experiments were performed twice

To further investigate the mechanisms underlying decreased STING phosphorylation and activation in patient cells and to clarify the functional role of the identified AMFR R594C variant on STING signaling, we went on to express Myc-tagged *AMFR WT* and the *AMFR R594C* variants in HEK293T cells together with FLAG-tagged STING and HA-tagged K27-ubiquitin. We then examined the impact of the AMFR variant upon STING K27-linked polyubiquitination in a two-step immunoprecipitation (IP) assay of STING. When we examined the immunoprecipitates by western blotting, we found markedly reduced STING K27-polyubiquitination in cells expressing *AMFR R594C* compared to *AMFR WT*-transfected cells, and this observation was consistent after the first as well as the second IP (Fig. 6A, B). Of note, we repeatedly observed slightly lower expression of the AMFR R594C variant compared to WT, although this was not detectable by normal western blotting of lysates from patient PBMCs (Fig. 2A, E), which may contribute to reduced signaling in the patient. When examining for STING trafficking from ER to Golgi in the HEK cell system using ImageStream (Fig. 6C), we found that expression of the AMFR R594C variant led to lower STING colocalization with Golgi compared to WT AMFR (Fig. 6D–E). Given that the patient is heterozygous for AMFR R594C, we next wanted to examine whether the patient's AMFR variant is exerting dominant negative activity upon AMFR WT with regards to STING phosphorylation and IFN-β production. To this end, we overexpressed *AMFR R594C* in HEK cells. These data show a dose-dependent inhibition of STING-driven *IFNB* induction upon expression of increasing amounts of the patient AMFR variant (Fig. 6F–G, Supplementary Fig. S3A).

Collectively, these data suggest that the AMFR R594C variant results in impaired STING ubiquitination, trafficking, and signaling, ultimately resulting in reduced antiviral IFN responses. Based on our biochemical characterization, we also propose the AMFR variant exerts a dominant negative activity on the function of the WT protein.

### Reconstitution of Patient PBMCs with AMFR WT Restores the cGAS-STING Pathway, ISG Induction, and Control of Viral Replication

**Fig. 7:**
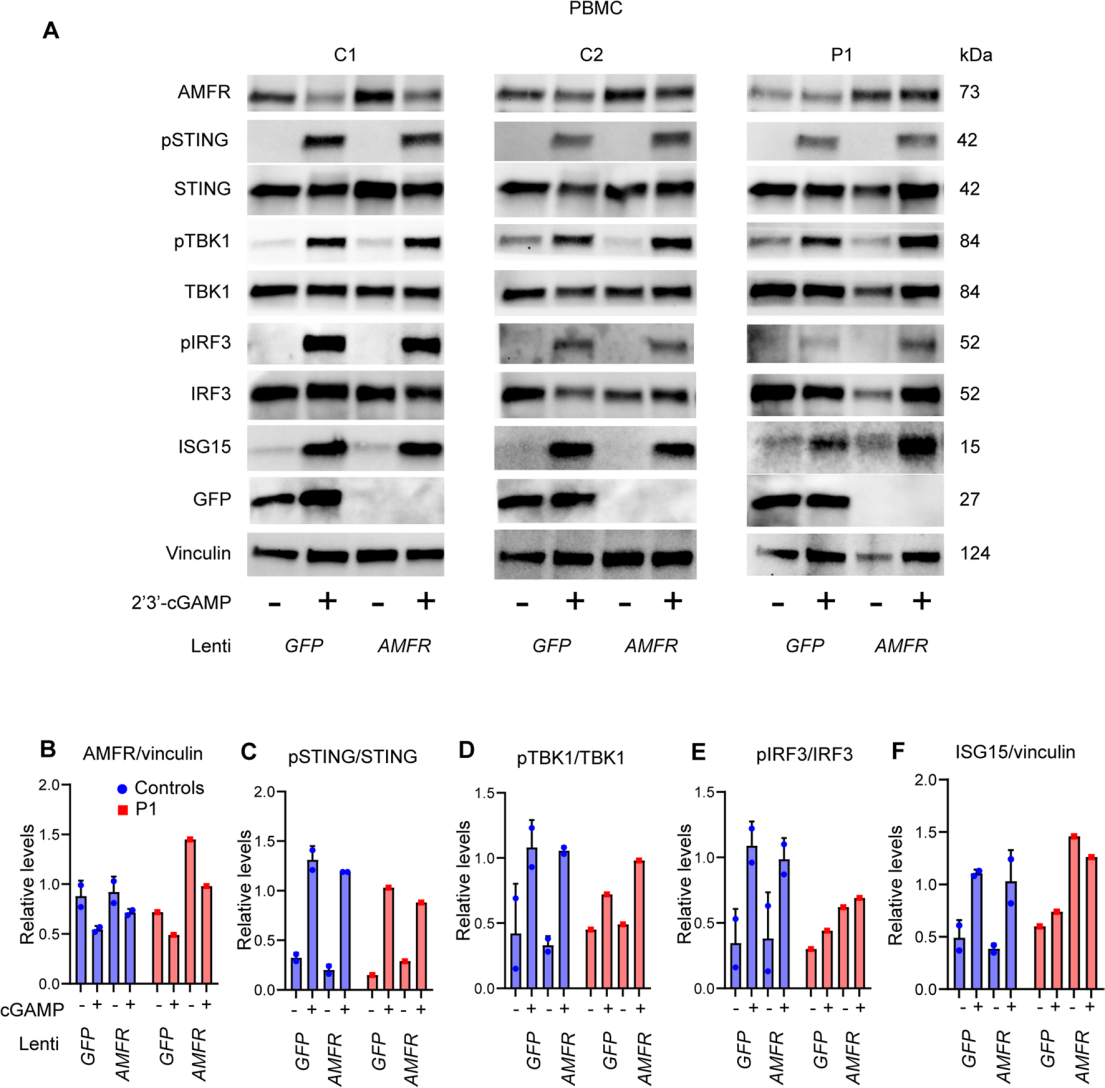
Reconstitution of patient PBMCs with WT AMFR restores cGAS-STING signaling and ISG responses. **A** Patient and control PBMCs were stimulated with 1.5 µg/mL PHA for 72 h and then transduced by VSV-G lentiviral vectors to express AMFR WT or GFP (control) (MOI 10). After 72 h, the cells were stimulated with 2′3′-cGAMP for 3 h, and cell lysates were analyzed by immunoblotting for AMFR, pSTING, STING, pTBK1, TBK1, pIRF3, IRF3, ISG15, GFP, and vinculin (loading control). **(B–F)** Quantification of the Western blot bands in (**A**)

In order to establish causality between the AMFR variant and the cellular phenotype, i.e., impaired STING phosphorylation and IFN responses) [36], we next reconstituted the patient PBMC with AMFR WT protein. To this end, we constructed VSV-G lentiviral vectors encoding GFP (control) or WT AMFR and first confirmed the efficiency of transduction of PHA-stimulated control PBMC (Supplementary Fig. S3B–D). Control and P1 PBMC were then transduced and stimulated with 2′3′-cGAMP. Importantly, the impaired ability of P1 PBMC to induce pTBK1 and pIRF3 in response to the STING-specific agonist was partly restored upon transduction with WT AMFR (Fig. 7A–H). Most strikingly, the AMFR transduced P1 PBMCs regained the ability to induce high expression of ISG15 2′3′-cGAMP stimulation, thus suggesting at least partial functional reconstitution of the cGAS-STING pathway (Fig. 7A, B). We did not observe a difference in levels of pSTING at the 3 h time point chosen, which could potentially be explained with delayed activation of STING in P1 GFP + cells, leading to observed equal levels of pSTING at the late time point post-stimulation despite lower net activation of STING. Notably, the P1 PBMC transduced WT AMFR regained the ability to induce IFN-stimulated gene expression upon 2′3′-cGAMP stimulation as demonstrated by expression of ISG15, thus suggesting functional reconstitution of the cGAS-STING pathway (Fig. 7A, F). Collectively, the reconstitution of patient PBMCs with WT AMFR partly reconstitutes the cGAMP-induced IFN response, suggesting that the patient AMFR variant disturbs antiviral immunity and ISG production.

## Discussion

The major finding of the present study is the description of a novel predisposition to severe viral infection involving abnormal AMFR-mediated polyubiquitination and decreased activation of STING in a child with a cellular phenotype showing impaired VZV-induced type I IFN responses and increased VZV replication and a clinical presentation with severe VZV infection and HLH. To our knowledge, this is the first report of a defect in STING signaling in humans causing increased susceptibility to viral infection.

Following the initial discovery of STING [37] and the subsequent discovery of cGAS [38], the cGAS-STING pathway is now appreciated as the major driver of the antiviral IFN response. Other DNA sensors, including RNA POL III, have been identified by many independent groups, but their respective roles in innate immunity are not fully clarified and may be dependent on cell type and cellular context [16, 39, 40]. cGAS recognizes foreign and host-derived dsDNA in a sequence-independent manner and catalyzes the formation of 2′3′-cGAMP that serves as a second messenger to the ER-bound adaptor protein STING [41, 42], which traffics to the Golgi, where it recruits TBK1, thus leading to phosphorylation of STING on serine 366, and this in turn leads to recruitment of IRF3 [43]. This positions IRF3 for phosphorylation by TBK1 allowing IRF3 dimerization, nuclear translocation, and induction of IFN-β gene expression [44]. The role of ubiquitination in STING biology is well documented and includes multiple types of ubiquitin linkage [45]. For instance, K63 ubiquitination by TRIM56, TRIM32, and Mul1 is important for the activation of signaling [46–48] and is targeted by HSV1 to facilitate CNS infection [49]. Moreover, K48 ubiquitination by RNF5 and RNF90 has been reported to promote STING degradation and downregulate signaling [50, 51]. Finally, K27-linked ubiquitination of STING by AMFR promotes STING ER-to-Golgi trafficking [35], which is a rate-limiting step in STING signaling [52] and promotes activation of the pathway and antiviral defense [35].

AMFR/g78 is an E3 protein ubiquitin ligase embedded in the ER membrane, important for the degradation of misfolded proteins in the ER-associated degradation response (ERAD). Upon microbial DNA challenge, AMFR catalyzes K27-linked polyubiquitination of STING, and this ubiquitin chain creates an anchoring platform for recruiting and activating TBK1, resulting in IRF3 phosphorylation and IFN-β induction [35]. Moreover, AMFR is also involved in regulating cholesterol biosynthesis which may impact ER membrane fluidity and STING trafficking from ER to Golgi, thereby influencing IFN activation and ISG responses [53]. Indeed, previous research has shown that STING signaling is linked to cholesterol metabolic pathways and cellular cholesterol content. Specifically, reprogramming of lipid metabolism leading to altered cholesterol synthesis via the mevalonate pathway can influence the threshold for type-I IFN production through STING activation [54]. The missense variant causing an amino acid substitution from arginine to cysteine is localized at the well-preserved position 594 within the G2BR domain, which is responsible for binding to the E2 ubiquitin-conjugating enzyme UBE2G, thus facilitating interaction between the RING domain and E2 enzyme and subsequently ubiquitin transfer. Deletion of the G2BR domain was reported to abolish the ERAD function of AMFR [55], and a pathogenic variant in this domain would therefore be expected to cause disturbed AMFR-mediated functions, most notably defective ubiquitination and activation of STING [35]. Indeed, we found that the cGAS-STING pathway is impaired in patient PBMCs stimulated by 2′3-cGAMP or dsDNA when measuring pSTING, pTBK-1, and ISG15, as well as decreased response to HSV1 despite the integrity of TLR3 and TLR9 signaling pathways in the patient. We were able to attribute this directly to decreased STING ubiquitination in cells expressing AMFR R549C as compared to cells expressing AMFR WT. Moreover, we demonstrate that the AMFR R549C variant interferes with AMFR WT in a partially dominant-negative manner with dose–response kinetics, although we cannot formally exclude a contribution from haploinsufficiency.

The effect of the AMFR variant may be through interaction with endogenous WT AMFR expressed from the other allele, thus eventually resulting in limiting amounts of AMFR for full STING activation. Alternatively, mutant AMFR may bind to STING and by competition cause dysregulation/insufficient activation of a fraction of cellular STING available for activation. At the molecular level, structural biology may provide some hypotheses. The R595C mutation in the patient AMFR variant is located in the E2 ubiquitin-conjugating enzyme binding domain responsible for interaction with the E2 ligase UBE2G2/UPC7 [56] and is part of a stretch of positively charged residues on positions 594–596. Therefore, we speculate that the R595C mutation impairs the recruitment of the E2 ubiquitin ligase to AMFR. Since AMFR is part of a larger protein complex including, for instance, INSIGR1, some likely mechanisms for the dominant negative effect include (i) more than one AMFR molecule is involved in each AMFR-containing complex, and the patient variant, therefore, leads to impaired recruitment of the E2 ligase to the complexes containing WT:mutant and mutant:mutant AMFR; (ii) there is only one AMFR molecule per complex, but other proteins in the complex are rate-limiting, and complexes containing mutant AMFR, therefore, compete for these proteins, hence exerting dominant negative activity toward complexes containing WT AMFR. Altogether, elucidation of the exact nature of the mechanism of the dominant negative activity required further investigation.

Finally, we establish the causal relationship between genotype and phenotype by demonstrating the reconstitution of the cellular phenotype (cGAS-STING signaling) in patient PBMCs complemented with WT AMFR. Whether the AMFR-STING defect is relatively specific to VZV and HSV1, or alternatively involves other DNA viruses remains to be resolved, but our finding of normal responses to SeV may indicate a defect mainly related to infection by DNA viruses. Collectively, the precise role of cGAS STING-mediated DNA sensing and IFN induction in humans remains to be determined.

The existence of defective innate DNA sensing predisposing to VZV infection, possibly through impaired signaling in response to the presence of viral cytosolic DNA, is similar to the previously reported IEI affecting another cytosolic DNA sensor RNA polymerase III, which has been described in children and adults with severe VZV CNS encephalitis, vasculopathy, and recurrent VZV meningoencephalitis [16–18]. Therefore, defective DNA sensing may be a common theme in VZV predisposition and might act in a partially cell-type-dependent manner. In the case of POL III deficiency, with CNS involvement constituting a prominent feature, we hypothesized that a particularly important role of POL III in recognizing the AT-rich VZV genome may be at play in certain cell types, such as neuronal cells, in which cGAS expression is low/absent [16, 17]. On the other hand, one study identified an important role of STING in mounting type I and type III IFN responses to VZV in human dermal fibroblasts and HaCaT keratinocytes with potential implications for varicella pathogenesis and suggesting an important role of cGAS-STING in the skin [57]. Recently, the cGAS-STING DNA sensing pathway was demonstrated to be required for IFN induction and VZV restriction during VZV infection in THP1 cells, and the VZV protein ORF9 was reported to antagonize cGAS-STING signaling and IFN production [58]. The observation that POL III dominates as a VZV sensor in mononuclear cells might in fact explain why we do not observe significantly decreased IFN induction in PBMCs in response to VZV and only could measure a modest increase in VZV replication in this cell type from the patient. The pattern of inheritance appears to be autosomal dominant with incomplete penetrance since the mother and two younger siblings also carry the AMFR R594C variant and so far have not experienced severe VZV infection. However, PBMCs from the mother of the patient also exhibit decreased phosphorylation of IRF3 (although less pronounced than in the patient), as well as significantly reduced IFNα/β responses to VZV infection together with increased VZV replication. The data suggest a milder cellular phenotype in the mother. However, since fibroblasts from the mother were not available, we were unable to make a precise comparison of antiviral responses in different cell types between patient, mother, and healthy controls. Indeed, fibroblast which produces more vivid antiviral responses than PBMCs may more precisely reflect the physiological situation in vivo. Moreover, it also has to be kept in mind that VZV infection models in primary human cells combined with the difficulty of measuring intracellular signaling pathways by a slowly replicating virus (i.e., not a potent agonist added at one given time) that can only be used in relatively low titers can be variable and is unlikely to give a completely straight biochemical picture in vitro regarding cellular responses. Incomplete penetrance is a frequent observation in IEI and may be due to influences from other genetic variants, differences in allele expression (for monoallelic variants), differences in viral load, or other immunomodulatory external factors. In order to hopefully avoid future severe illness in the siblings, they have been vaccinated against VZV by the live attenuated VZV vaccine.

As to the possible link between this variant causing increased VZV replication and severe VZV infection to the development of HLH, this remains insufficiently understood. However, some previous observations in the literature may suggest a possible pathophysiological mechanism. Among cases of secondary HLH, viral infection is the most common trigger [59], and more recently, it has been suggested that monoallelic variants in known HLH genes, or less penetrant other gene variants associated with IEI, may underlie HLH [26, 60]. This may be the case for other IEI affecting innate immunity as exemplified by a previous study demonstrating disseminated VZV and HLH in a patient with GATA2 defect [23]. In this context, enhanced viral replication and an increased load of PAMPs may increase the risk of developing a hyperinflammatory state. Further, we cannot exclude that dysregulated STING activation as described in our patient may lead to hyperinflammation during a high viral burden. We suggest that the variant may not have a very strong direct disease-causing effect but rather lowers the threshold for virus-induced inflammation and HLH. The link between STING-signaling and reduced threshold for HLH is further underpinned by a recent study implicating the well-established HLH-disposing gene *UNC13D* in STING regulation [61]. Collectively, these complex cellular interactions between pathogen/PAMP and the immune system that may trigger a cytokine storm and HLH cannot be sufficiently mimicked and studied in an in vitro system. Based on the present report, we would encourage others with the relevant expertise to investigate this matter in a physiological context, which would however require modified animals, since VZV is strictly a human pathogen.

Previously, Goldbach-Mansky and associates described a vascular and pulmonary syndrome in patients with gain-of-function variants in the STING-encoding gene *STING1* (formerly known as *TMEM173*) and suggested the name STING-associated vasculopathy with onset in infancy (SAVI) for this autoinflammatory interferonopathy [62]. The authors identified three different mutations in exon 5 of *STING1* in these six patients and observed constitutive STAT1 phosphorylation and increased constitutive and inducible type I IFN expression in patient cells [62]. Since then, several other publications have described additional patients and extended the clinical phenotype [63, 64]. Moreover, other IEIs also affecting IFN production, such as STAT2 deficiency, translate into partially overlapping clinical manifestations together with the interferonopathy signature [65, 66]. However, despite the description of numerous IEIs affecting pathogen and PAMP receptors and their downstream IFN-inducing signaling pathways causing susceptibility to severe viral infections, defects in the cGAS-STING pathway in humans have not been previously reported. The clinical and cellular phenotype described here is significantly different from the one ascribed to SAVI, and notably, we did not observe increased IFN nor ISG levels in vitro under the given conditions examining patient cells harvested after acute illness. However, we cannot entirely exclude that elevated ISGs may have been present in the circulation of the patient during the acute episode, and thus we cannot rule out a degree of autoinflammation/interferonopathy caused by the AMFR variant and possibly associated to the development of VZV-triggered HLH in the patient.

In conclusion, we suggest a novel genetic etiology of severe VZV disease in childhood, also representing the first IEI related to a defect in the STING-cGAS pathway. The present work contributes information on the question of the role of DNA sensors in host defense in human immunology. Together with our previous work on POL III, the available data suggest that POL III and cGAS-STING may each play non-redundant roles in VZV immunity in humans, depending on the cell type and tissue involved. Collectively, the precise role of cGAS STING-mediated DNA sensing and IFN induction in humans remains to be further studied. Identification of human IEIs involving these molecules remains a powerful tool for establishing the contributions of relevant immune signaling pathways in humans and for gaining valuable insights into how dysregulated cGAS-STING signaling may lead to human disease, ranging from autoinflammatory interferonopathy to severe viral infection.

### Supplementary Information

Below is the link to the electronic supplementary material.

Supplementary file1 (DOCX 689 KB)

## Data Availability

All data related to this study will be shared at the request of other investigators for purposes of replicating procedures and results, according to national and international GDPR rules and following individual DTA and MTA rules with relevant investigators.
