# Supplementary material for: Impaired STING Activation Due to a Variant in the E3 Ubiquitin Ligase AMFR in a Patient with Severe VZV Infection and Hemophagocytic Lymphohistiocytosis
Source: J Clin Immunol. 2024 Jan 26;44(2):56. doi: 10.1007/s10875-024-01653-5 (PMC10817851; doi:10.1007/s10875-024-01653-5)
Supplement: Supplementary file 1 — Supplementary file1 (DOCX 689 KB) [file 10875_2024_1653_MOESM1_ESM.docx]

**Supplementary Material**

***Patient case and paraclinical findings***

The patient is a 3-year-old boy of White European ancestry born from parents in a non-consanguineous marriage. Following exposure in day-care he developed varicella. After two to three days of what seemed to be an uncomplicated infection, the condition worsened. The temperature raised to around 40^O^C. New lesions appeared and both these and existent lesions enlarged, became deeper and took a red-purple color. Oral acyclovir was initiated. Due to intermittent altered consciousness, suspected of being febrile delirium, and tachypnea, he was admitted to hospital. Based on the clinical presentation with myriads of large, hemorrhagic and necrotic varicella lesions with a universal distribution, bilateral infiltrates on chest X-ray, requirement for supplemental oxygen and slightly elevated liver enzymes, the boy was diagnosed with severe, probably disseminated, varicella infection and suspected of a complicating bacterial sepsis. Intravenous high dose acyclovir and cefuroxime were initiated and the boy was transferred to the tertiary center. The initial air-way specimen was VZV PCR positive and no other etiologic agent were identified on any PCR or culture. Lumbar puncture performed after transferal was without pleocytosis (less than 5 x 10^6 cells/L), showed normal glucose and normal protein content (3.0 mmol/L and 0.24g/L respectively) and was PCR negative for herpes simplex virus (HSV) type 1 and 2, CMV, EBV and VZV in the cerebrospinal fluid (CSF). Activated monocytes were observed by Giemsa staining of the CSF. Due to extensive and progressive systemic inflammation, elevated ferritine, triglyceride, cytopenia, splenomegaly and fever together with presence of hemophagocytosis in the bone marrow, HLH (based on the 2004 HLH criteria, se supplementary table S2) was considered likely and due to the unstable condition it was decided to initiate treatment according to the HLH-2004 protocol. A routine clinical immunological evaluation was performed (supplementary table 2) which revealed that the patient has normal perforin levels, normal NK and T cell degranulation as well as normal NK cell cytotoxicity, thus arguing against a defect in cytotoxic lymphocyte function. The patient had decreased concentrations of NK and B cells in whole blood, which most likely could be ascribed as a secondary feature to HLH [32, 33]. Subsequently, WES was performed using a gene panel comprising 572 PID and HLH associated genes, together with all known genetic etiologies of HSV and VZV infection (as per May 2023) which did not identify any known disease-causing variants. The patient was considered for bone marrow transplantation, due to the concern that primary HLH could be the case despite absence of gene defects related to fHLH. The boy had not previously demonstrated increased susceptibility or severity to infectious diseases and had been following the Danish routine childhood vaccination program, including live MMR vaccination at age 15 months without major side effects. He made a slow but good recovery, was discharged after one month but did not show full remission and required long-standing maintenance therapy. Finally, HLH-treatment was discontinued after 40 weeks. But acyclovir was continued for prophylaxis. Repeat bone marrow examinations continued to demonstrate hemophagocytosis, even at the time where HLH therapy was discontinued. Only four months later the last bone marrow examination was completely normal. Varicella lesions healed slowly and with marked circular scaring. Intermittent episodes with erythema in the periphery of several lesions followed, and although VZV was not identified following the initial disease, “inflammatory infiltrate as seen with varicella” was described in a skin biopsy taken from one of these flares a few weeks after HLH therapy was discontinued. Serological analyses have shown that the patient was anti-VZV and anti-HSV 1/2 negative as of March 2017, whereas the mother was anti-VZV positive in 2017. The two younger siblings were both VZV vaccinated with initial dose at age 9 month; HSV and VZV serology do not exist for these. The mother was reported to have experienced varicella infection in childhood within the normal range of disease presentation without hyperinflammation or any specific sequelae, although she had an otherwise inconspicuous case of shingles as an adult.

**Supplementary Figures**


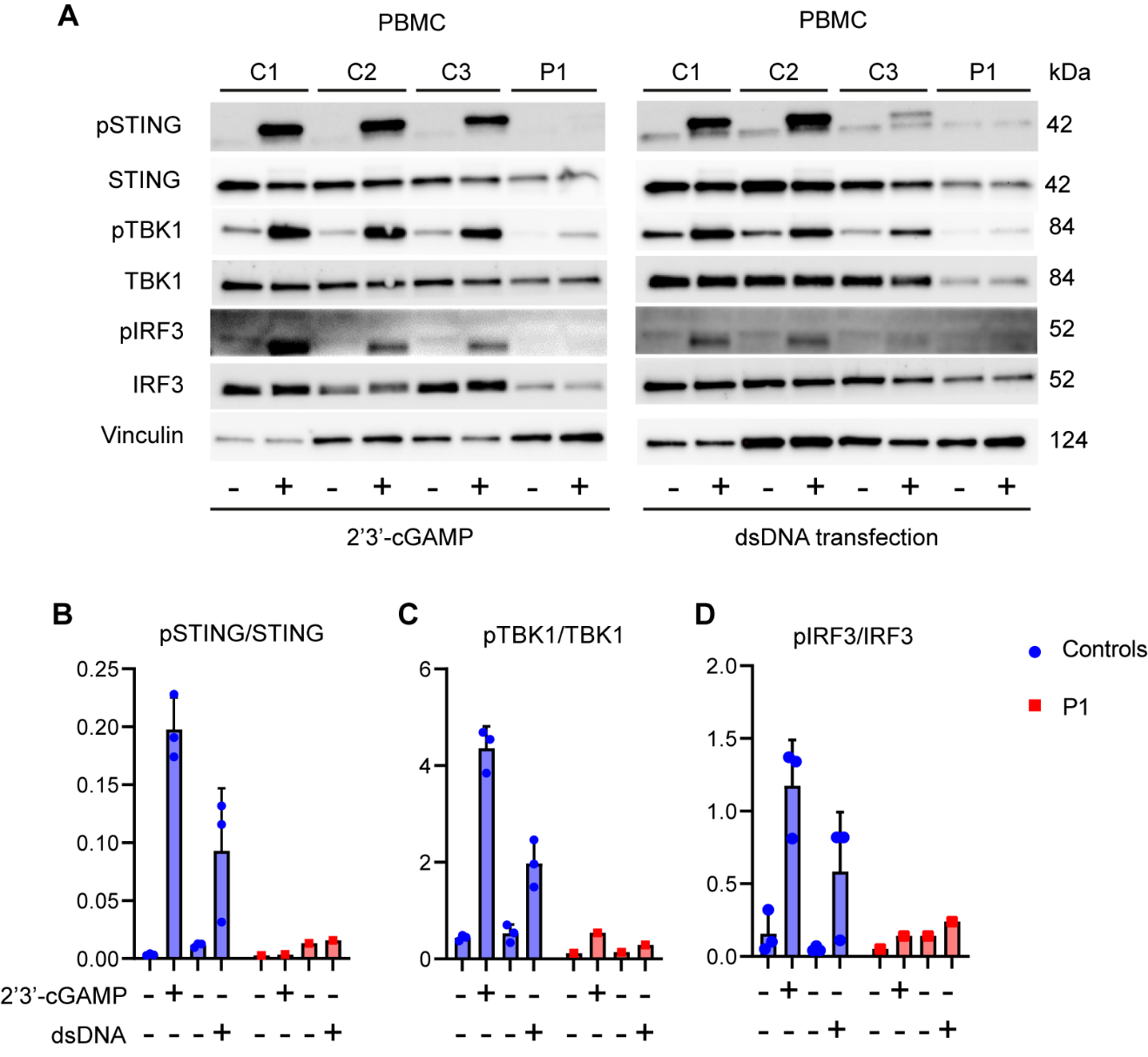


**Supplementary Figure 1. Reduced STING signaling in patient PBMCs in response to 2’3-cGAMP and dsDNA stimulation.** PBMCs from the patient and three healthy controls (C1-C3) were stimulated with **(A)** 100 ug/mL of 2’3’-cGAMP or 2 ug/mL dsDNA transfected with lipofectamine or lipofectamine as mock for 3 h followed by lysis for Western blotting to measure the expression levels of pSTING, STING, pTBK1, TBK1, pIRF3, IRF3, and vinculin (loading control). **(B-D).** Quantification by densitometry.


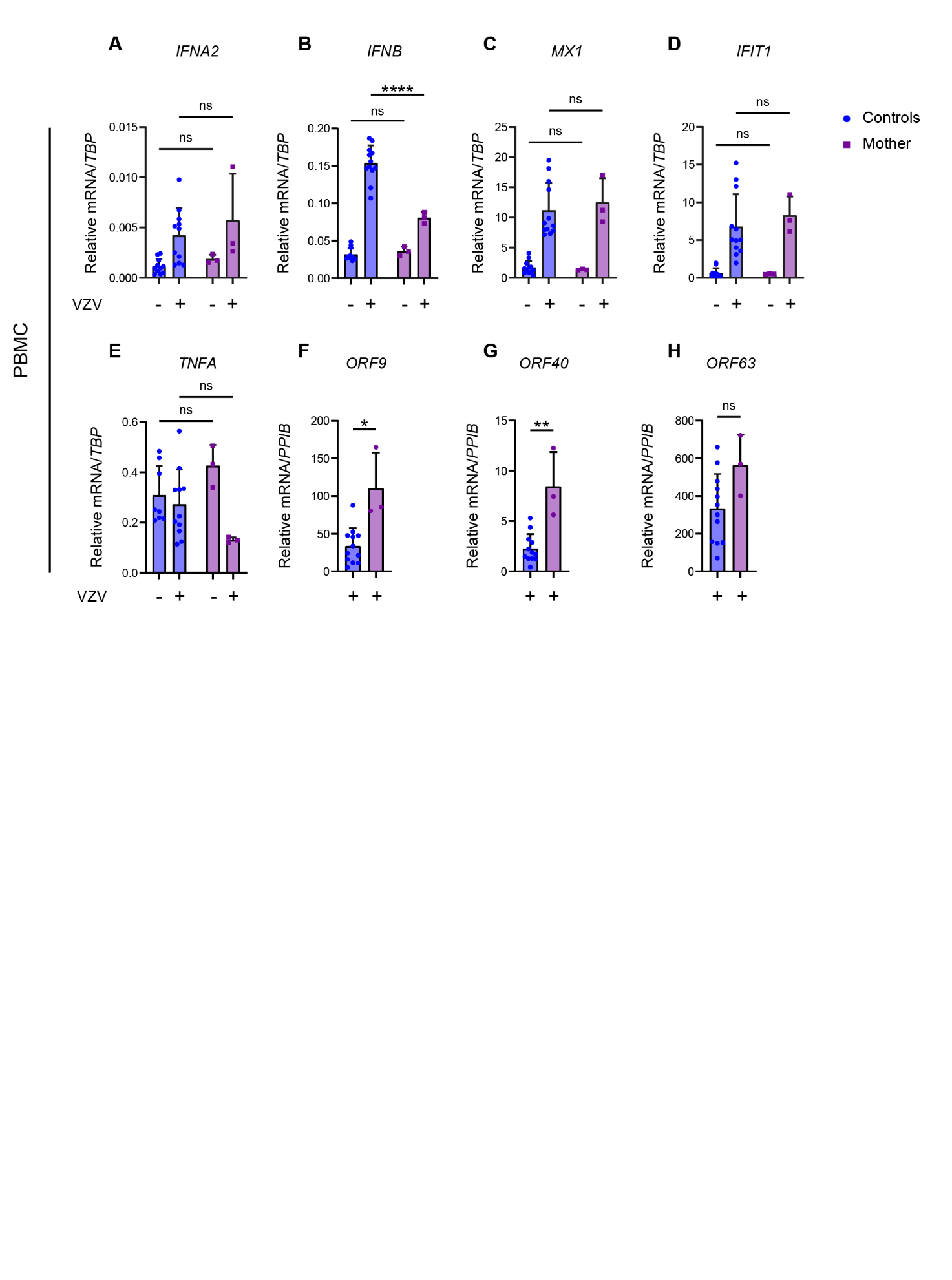


**Supplementary Figure 2**. VZV infected PBMCs were lysed at 24 hpi, and RNA was purified and subjected to RT-qPCR for the expression of *IFNA2*, *IFNB*, *MX1* and *IFIT1* (A-E) as well as VZV open reading frame gene (ORF)9, ORF40, and ORF63 (F-H). IFN and ISG were normalized to TBP. ORF mRNA was normalized to PPIB levels, and statistics calculated using a 2-way ANOVA and Sidak’s multiple comparisons test.


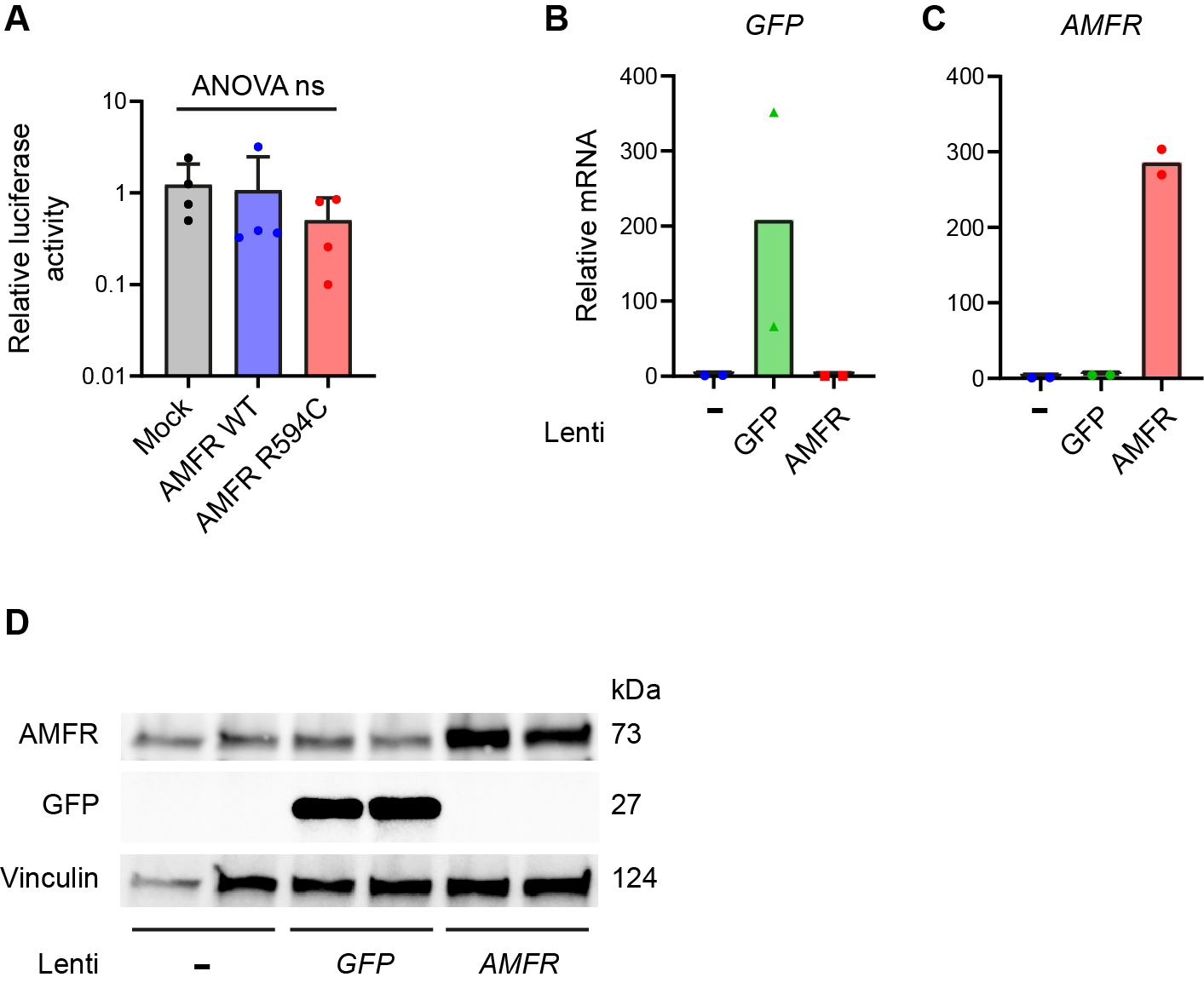


**Supplementary Figure 3: Lentiviral transduction efficiency of PHA-stimulated PBMC.**

**(A)** HEK 293T cells with stable STING expression were transfected with 50ng WT AMFR, R594C AMFR, or empty vector together with IFNB1 promoter luciferase and β-actin Renilla reporters. Cells were lysed 18 h later and reporter gene activity was measured. **(B-D)** PBMC were pre-stimulated with 1.5 μg/mLPHA for 72 h before transduction with VSV-G lentiviral vectors encoding GFP (control) or WT AMFR. **(B, C)** Cells were lysed for RT-qPCR for mRNA levels of *GFP* and *AMFR*. **(D)** Cells were lysed for western blotting for AMFR, GFP, and vinculin (loading control).

**Supplementary tables**

| **Gene** | **Transcript ID** | **Transcript Variant; Protein Variant** | **Translational**  **impact** | **CADD; MSC scores** | **SIFT Function Prediction** | **gnomAD frequency (%)** | **dsSNP ID** |
| --- | --- | --- | --- | --- | --- | --- | --- |
| ***ECM1*** | NM_004425.4; | c.135_136insGC; p.P46fs*133 | Frameshift | 24.800; 0.001 | NA | - | - |
| ***ITGB8*** | NM_002214.3 | c.1198T>G; p.Y400D | Missense | 15.610; 3.313 | Tolerated | 0.002 | 778043359 |
| ***EGR2*** | NM_000399.5 | c.644C>T; c.494C>T; p.T215M | Missense | 21.100; 1.828 | Tolerated | 0.063 | 139147487 |
| ***NECTIN1*** | NM_002855.5 | c.1331_1333dupAGG; p.E444dup | in-frame | 16.670; NA | NA | - | 753988307 |
| ***BRIP1*** | NM_032043.2 | c.139C>G; p.P47A | Missense | 25.200; 0.001 | Damaging | 0.025 | 28903098 |
| ***PRKCA*** | NM_002737.3 | c.556A>G; p.M186V | Missense | 24.500; 3.313 | NA | 0.010 | 200732611 |
| ***MYO5B*** | NM_001080467.3 | c.5449A>G; p.M1817V | Missense | 18.940; 0.017 | Activating | 0.006 | 200172993 |
| ***RNASEH2A*** | NM_006397.2 | c.518C>T; p.P173L | Missense | 24.400; 10.51 | Damaging | 0.003 | 369355807 |
| ***PIK3R2*** | NM_005027.4 | c.1938C>T; p.I646I | Synonymous | 19.070; 23.5 | NA | 0.068 | 146987351 |
| ***LRBA*** | NM_006726.4 | c.1903_1905delGGT; p.G635del | in-frame | NA; 23.5 | NA | - | - |

**Supplementary Table S1. Additional genetic variants identified in the patient by whole exome sequencing and not ascribed a disease-causing potential.** The listed gene variants identified within the following search criteria: CADD score >15 and >MSC, GnomAD frequency <0.1% and possible biological relevance together with all genes related to known IEI according to the IUIS Guidelines. CADD= Combined Annotation Dependent Depletion, MSC= Mutation significance cutoff, SIFT= Sorting Intolerant From Tolerant, gnomAD= The genome aggregation database, NA= Information not available. - = variant not reported in gnomAD.

| Cell subtypes and immunoglobulin values | Patient value | Normal value |
| --- | --- | --- |
| Total lymphocytes | 0.3*10^9^ cells/L | 1.10-9.90*10^9^ cells/L |
| CD3- CD56+ (NK) cells | 0.01*10^9^ cells/L | 0.05-0.85*10^9^ cells/L |
| CD3+ (T) cells | 1.26*10^9^ cells/L | 1.95-6.70*10^9^ cells/L |
| CD3+ CD4+ (T) cells | 0.79*10^9^ cells/L | 0.7-2.5*10^9^ cells/L |
| CD3+ CD8+ (T) cells | 0.44*10^9^ cells/L | 0.2-1.20*10^9^ cells/L |
| CD19+ (B) cells | 0.07*10^9^ cells/L | 1.10-9.90*10^9^ cells/L |
| NK cell degranulation (CD107a)^a^ | 24% | 19%^b^ |
| T cell degranulation (CD107a)^c^ | 77% | >5% |
| NK cell-mediated cytotoxicity^d^ | 31 lytic units | >10 lytic units |
| IgG | 6.5 g/L | 4.1-12.0 g/L |
| IgM | 0.25 g/L | 0.30-1.84 g/L |
| IgA | 0.48 g/L | 0.09-1.37 g/L |

**Supplementary Table S2. Results from clinical Immunological investigations of the patient during hospital admission.**

^a^:NK cell (CD3- CD16+ CD56+) degranulation was measured as the proportion of CD107a on the surface of NK cells following stimulation with K562 lymphoblast cell line.

^b^:Measured in an age-matched healthy control.

^c^:T cell (CD3+) degranulation after stimulation with anti-CD3 antibodies.

^d^:NK cell (CD3- CD56+)-mediated cytotoxicity on the K562 lymphoblast cell line.

| **Diagnostic criteria based on HLH-2004 protocol** | **at least 5 of 8** |
| --- | --- |
| Fever (peak temperature of > 38.5° C for > 7 days) | **Yes** |
| Splenomegaly (spleen palpable > 3 cm below costal margin) | **Yes**  Splenomegaly also confirmed on MRI longest diameter 10.4cm at age 3y6m |
| Cytopenia involving > 2 cell lines (hemoglobin < 9 g/dL [90 g/L], absolute neutrophil count < 100/mcL [0.10 × 10^9^/L], platelets < 100,000/mcL [100 × 10^9^/L]) | Isolated thrombocyto­penia in the range 30-90 x 10^9^/L |
| Hypertriglyceridemia (fasting triglycerides > 177 mg/dL [2.0 mmol/L] or > 3 standard deviations [SD] more than normal value for age) or hypofibrinogenemia (fibrinogen < 150 mg/dL [1.5 g/L] or > 3 SD less than normal value for age) | **Yes**  Hypertriglyceridemia (fasting triglycerides 3.54 mmol/L; fibrinogen 211 mg/dL) |
| Hemophagocytosis (in biopsy samples of bone marrow, spleen, or lymph nodes) | **Yes**  Hemophagocytosis identified In bone marrow (present during acute disease and on repeated re-examinations including nine month following initiation of HLH therapy, disappeared on re-examination after further four months) |
| Low or absent natural killer cell activity | **No**  NK cells almost absent, but normal degranulation and cytotoxicity |
| Serum ferritin > 500 ng/mL (> 1123.5 pmol/ng/mL) | **Yes**  Above 40,000 ng/mL |
| Elevated soluble interleukin-2 (CD25) levels (>2400 U/mL or very high for age) | Likely  CD25 measurement not unavailable, but CD163 was elevated to 4,250 μg/L. |

**Supplementary Table S3. Patient laboratory values compared to HLH criteria**
